# Working on differentiated nursing practices in hospitals: A learning history on enacting new nursing roles

**DOI:** 10.1111/jan.16240

**Published:** 2024-05-29

**Authors:** Dieke Martini, Hugo Schalkwijk, Lisette Schoonhoven, Mirko Noordegraaf, Pieterbas Lalleman

**Affiliations:** ^1^ Research Group for Person‐Centeredness in an Ageing Society Fontys University of Applied Sciences Eindhoven The Netherlands; ^2^ Julius Centre for Health Sciences and Primary Care University Medical Centre Utrecht, Utrecht University Utrecht The Netherlands; ^3^ Utrecht School of Governance Utrecht University Utrecht The Netherlands

**Keywords:** context, differentiated nursing practices, learning history, nurse staffing, nursing roles, nursing work, participatory action research, skill mixes

## Abstract

**Objective:**

This study aims to better understand how new future‐oriented nursing roles are enacted in a general hospital.

**Design:**

A learning history, that is, a participatory action‐oriented research design to explore and foster organizational learning.

**Methods:**

Data collection consisted of a (historical) document analysis, the shadowing of differentiated nursing practices (36 h), 22 open interviews, 4 oral history interviews, 2 focus groups and a podcast series (7 h) created with participants.

**Results:**

The data gathered revealed three important themes regarding enacting new nursing roles: (1) stretching the nature of nursing work, (2) using earlier experiences and (3) collectively tackling taboos.

**Conclusions:**

Differentiated nursing practices and enacting new nursing roles have long and complex histories. Attempts to differentiate are often met with resistance from within the nursing profession. This study shows how the new role of *nurse coordinator* was negotiated in nursing teams. With a bottom‐up approach focused on collective responsibilities. By acknowledging and reflecting on the past, spaces were enacted in which the role of nurse coordinator became one role, among others, in the delivery of patient care.

**Impact:**

This study provides an innovative perspective on differentiated nursing practices by focusing on the past, the present and the future. We found that local, situated conditions can be taken as starting points when new nursing roles are enacted. In addition, shifting focus from individual nursing roles to nursing team development, emphasizing collective responsibilities, softens strong (historically) grown emotions and creates spaces in which new roles become negotiable.

**Patient or Public Contribution:**

No patient or public contribution.

## 1 INTRODUCTION

Increased healthcare costs, an ageing population, growing healthcare needs and care complexity put pressure on healthcare systems (World Health Organization, 2020). Nurses play an essential part in facing these challenges as front‐line providers of healthcare. Research shows that higher registered nurse staffing likely leads to better patient care (Twigg et al., 2019). However, an impending worldwide shortage of nursing staff jeopardizes this (World Health Organization, 2020). Many nurses leave the profession prematurely due to growing professional dissatisfaction (Bahlman‐van Ooijen et al., 2023; Lartey et al., 2013), in addition, an insufficient number of new nurses enter the profession (Van Kraaij et al., 2021; Van Schothorst‐Van Roekel et al., 2020). To prevent nursing shortages effectively, healthcare organizations aim to invest in career development and the improvement of the nursing work environment (World Health Organization, 2020). Differentiated nursing practices, in which nurses are encouraged to take on challenging roles that match their educational backgrounds, experiences and expertise, are seen as a possible way to attract and retain registered nursing professionals (Boston‐Fleischhauer, 2019; Matthias, 2015; Van Kraaij et al., 2021).

In addition to preventing nursing shortages, differentiated nursing practices could benefit patient care. Extensive international longitudinal research indicates that healthcare organizations with higher registered nurse staffing have significantly better patient outcomes (Harrison et al., 2019; Tuinman et al., 2021; Twigg et al., 2019). However, the introduction of new nursing roles is hampered by a persistent, historically formed narrative of nursing as bedside work aimed at direct patient care. Emphasis is put on technical and practical skills. (Hallam, 2002; Rafferty, 2018). This narrative enforces ideas about what nursing work is and what it is not (Boston‐Fleischhauer, 2019; Carryer, 2019; Felder et al., 2022; Rafferty, 2018). In order to address and/or adapt this narrative, the literature suggests that it is important to reflect on its past. Reflection helps to create joint futures for the profession (Smith, 2020).

In addition, much is still unknown about the complexity of enacting new nursing roles in the daily practices of nurses (Van Schothorst–van Roekel et al., 2021). Changes in nurse staffing levels and skill mixes – that is, the combination of different categories of nursing staff employed to provide patient care (Aiken et al., 2003) – should be carefully investigated. Such changes can best be determined locally before being ‘routinely implemented’ in healthcare organizations (Backhaus et al., 2021; Griffiths, 2014, p. 354). However, research concerning differentiated nursing practices and enacting new nursing roles often does not address either the local contexts into which new roles are introduced or the complex histories of differentiated nursing practices.

## 2 BACKGROUND

### 2.1 Differentiated nursing practices: A resurfacing debate

Nurses have supported or opposed differentiated nursing practices internationally (Boston‐Fleischhauer, 2019; Felder et al., 2022; Matthias, 2015; Rafferty, 2018). For example, this discussion has been ongoing in the United States for over 100 years since the introduction of the Bachelor of Nursing (BN) programme in 1916 (Matthias, 2011). The concept is at odds with the mindset that ‘*a nurse is a nurse*’ (Boston‐Fleischhauer, 2019 p291) and collides with a strong public perception that ‘*nurses do not need degrees to be good nurses, they only need to be kind to care*’ (Rafferty, 2018, p. 479). Work that is done by nurses away from the bedside, for example, organizing work, scientific work and quality improvement or work in political arenas, is not recognized as nursing work (Allen, 2014; Van Wijk et al., 2022; Verhoeven, Marres, et al., 2023). This is problematic for the development of new nursing roles, specifically for bachelor‐trained nurses, because they distinguish themselves mainly in the areas of organizing care and quality improvement (Van Schothorst–van Roekel et al., 2021).

### 2.2 Nursing education in the Netherlands

In Dutch healthcare, differentiated nursing practices have been a topic of discussion ever since the first bachelor‐trained nurses entered Dutch healthcare organizations in the 1970s. Historically, the Dutch nursing profession had five different entry routes. Prior to 1972, care institutions offered in‐service training programmes. Nurses were trained in (1) general hospital nursing, (2) psychiatric nursing or (3) disability nursing. These programmes were hands‐on and aimed at direct patient care. These programmes were akin to diploma degrees internationally. In 1972, two new educational pathways were introduced to align with the Netherlands' educational structure: (4) vocational training and (5) bachelor training (Schalkwijk et al., 2024). Temporarily, all five routes existed simultaneously. However, currently, the 4‐year full‐time vocational and bachelor programmes prevail.

The vocational training programme is offered at the non‐university secondary vocational education level. It emphasizes practical bedside care. The bachelor's training programme is offered at the higher professional education level. It focuses on theory‐based practice education and resembles the international bachelor of nursing programme. However, the bachelor programme is not integrated into traditional universities but is offered by universities of applied sciences (Schalkwijk et al., 2024). In 1993, the Dutch National Professions in Individual Healthcare Act was enacted to protect professional titles and regulate the work of healthcare workers' work individually. Under this act, *graduates of all five educational pathways* could apply for the title of ‘Registered Nurse’ (Schalkwijk et al., 2024). Consequently, graduates from the five different entry routes work as registered nurses in Dutch Healthcare organizations.

Strikingly, there is no formal (legislated) distinction in work, wages, responsibilities, nurse‐to‐patient ratio or activities of registered nurses based on educational background (Felder et al., 2022; Van Kraaij et al., 2021). Failed attempts to differentiate nurse staffing levels and skill mix in the Netherlands over the years accumulated in a ‘last resort’ proposed statutory amendment in 2019 (Felder et al., 2022). Differentiation between bachelor and vocationally trained nurses would be created by law, allowing for a formal distinction in the organization and design of nursing work (Van Kraaij et al., 2021). Bachelor‐trained nurses would receive the title of nurse coordinator, and vocationally trained nurses the title of *Nurse*. A national expert committee drafted two job profiles in which particular responsibilities within a wider range of nursing work (e.g. direct patient care, organizing care activities within and across wards, engaging in quality improvement initiatives and coaching and implementing evidence‐based practice (EBP)) were specified per role (Terpstra Committee, 2015). These job profiles formed the basis for developing new nursing roles in practice, leading to a change in nurse staffing levels and skill mix at the wards (Van Schothorst–van Roekel et al., 2021). However, nurses who fell outside the amendment's scope – for example, vocationally trained nurses and in‐service trained nurses – felt ‘devaluated’ and ‘depreciated’ because of subsequent salary differences and the tasks and responsibilities that bachelor‐trained nursing colleagues would perform. Under heavy protests from groups within the profession, emphasizing the fact that nurses are ‘all equal’ and all work ‘at the bedside’, the proposed amendment was withdrawn (for an overview of the protests, see Felder et al., 2022). Nevertheless, hospitals continue introducing new nursing roles in their organizations (Van Kraaij et al., 2021).

This study focuses on a general hospital (766 beds) that provides tertiary care where the nurse coordinator role is introduced despite national and local protests.

## 3 THE STUDY

### 3.1 Aim

In order to enhance nursing and organizational capabilities to deal with changing client and care circumstances, this study aims to better understand how new future‐oriented nursing roles are enacted in a general hospital.

## 4 METHODS

### 4.1 Design

We used a learning history method (Roth & Kleiner, 1995). This participatory action‐oriented research approach is designed to ‘explore and foster learning in organizations’ by working closely with organizations and professionals (Lyman & Moore, 2018). As such, it helped us draw out experiences, successes and tensions around introducing and enacting a new nursing role, nurse coordinator, for bachelor‐trained nurses in a Dutch general hospital.

### 4.2 Theoretical framework

Learning histories are developed to actively stimulate organizational learning by focusing on the past, present *and* future (Lyman & Moore, 2018; Roth & Kleiner, 1995). In a learning history, a *joint narrative* of both researchers' and practitioners' learning efforts is composed using the rules and methods of qualitative research (Bradbury‐Huang, 2010). As is customary in action research, the narrative is composed in collaboration with a group of practitioners. The narrative stimulates recognition, reflection and progress, allowing organizational members to recognize their own story and become familiar with that of others (Keulen & Kroeze, 2012). Learning histories thus *actively stimulate* organizational learning and stimulate the formulation of new shared futures (Lyman & Moore, 2018). We shared the learning history we created within the hospital and nationally in a podcast (Martini et al., 2021a) and a written learning history report (Martini et al., 2021b). Both aimed to enrich conversations on differentiated nursing practices.

### 4.3 Study setting and recruitment

This study is part of a national government‐funded research programme called RN2Blend, which investigates and accompanies differentiated nursing practices in the Netherlands (Lalleman et al., 2020). This particular study occurred in a Dutch general hospital (766 beds) providing tertiary care. At the time of this study, the hospital introduced the role of nurse coordinator for bachelor‐trained nurses and nurses who demonstrably functioned at the bachelor level. Both *nurses* and *nurse coordinators* drafted, executed and coordinated the overall nursing care process based on the individual patient to meet the care needed or demanded. In addition, the nurse coordinator was expected to improve the quality and organization of nursing care from a coherent and overarching perspective. Furthermore, the nurse coordinator was expected to take on a coaching and initiating role in developing, implementing and safeguarding improvements in the quality of patient care and in promoting expertise within the nursing domain. The job description implied a hierarchy in which nurse coordinators would provide functional guidance to nurses in the above‐stated areas (internal documents). Aspiring nurse coordinators were expected to develop their new roles in the nursing wards' daily practices.

In line with the learning history method, a research team was formed. This team included both researchers from outside the hospital and internal researchers. The internal researchers were (1) the project lead responsible for the introduction of differentiated nursing practices, (2) the chair of the nursing council, (3) a nurse scientist and (4) an educational advisor. We teamed up to collect data and create a learning history that would stimulate conversation within the hospital on the new nursing roles that were introduced. The small group of internal researchers participated in the formulation of a general topic for the learning history, the selection of participants, the data analysis and the data dissemination. Participants for the study were selected using purposeful sampling, either on recommendation from inside researchers or based on encounters while shadowing.

### 4.4 Data collection and participants

Data were collected over a period of 6 months (August 2020 to January 2021) using multiple data‐gathering methods. The different methods enabled both the researchers and the participants, in various ways, to narrate their experiences around events that were related to differentiated nursing practices. The data consisted of:

#### 4.4.1 Documents

An archival document analysis of the history of differentiated nursing practices in the Netherlands was conducted by the second author (HS), a historian on the research team. These documents included nurses' professional profiles and government and policy documents. Combining these sources with existing literature on the history of nurse legislation (see, e.g.: Van Der Peet, 2021), we drew up a timeline, providing a historical overview of attempts to differentiate nursing practices at the government level.

#### 4.4.2 Shadowing

The first author [DM] shadowed differentiated nursing practices for a total period of 36 h to observe and capture practices that are difficult to describe in words (Czarniawska, 2007). Usually, specific participants are followed to learn more about their world ‘from the inside’ (De Kok et al., 2023; Lalleman et al., 2017; Oldenhof, 2017). In this instance, we chose to shadow differentiated nursing practices as a ‘quasi‐object’ (Czarniawska, 2007; Oldenhof, 2017). Quasi‐objects are entities that, unlike people or objects, can take different shapes in different places. For example, it can be a point on the agenda in a meeting or a policy document. This allowed us to observe and follow differentiated nursing practices throughout the hospital, for example, during meetings with middle managers, the hospital's academy, the nursing council, team meetings, etc.

#### 4.4.3 Interviews

Two members of the research team [DM, PL] conducted 22 open interviews (45–90 min) from an interpretive perspective (Langley & Meziani, 2020). Participants were selected using purposeful sampling based on observations from inside researchers or encounters while shadowing. We invited the participants to share their opinions, troubles, hopes and thoughts on differentiated nursing practices and nursing work. In addition, four *oral history interviews* were conducted by the second author (HS) to complement the archival data. In oral history interviews, participants are asked to reflect on past experiences that are not officially archived and often left untold (Portelli, 2009). From the document analysis, we learned that there had been earlier attempts to differentiate in the hospital. The oral history interviews helped us to learn more about nurses' experiences of these earlier attempts. This enriched our understanding of why these earlier attempts had failed. These four respondents were selected through purposeful sampling, specifically aiming at a representation of experiences of both pro and opponents of differentiated nursing practice (Boschma et al., 2008). All interviews were conducted in person or online via MS Teams, audio recorded and transcribed verbatim. Participants included nurses (13), (middle) managers (6), medical specialists (3), board members (2), the Chief Nursing Information Officer and an HR professional.

#### 4.4.4 Focus groups

Two focus groups (of, respectively, 50 min and 85 min) were conducted. In the first focus group, four nurses who worked as nurse coordinators and an educational advisor participated. All participants were invited to share how they experienced their (new) roles. This provided us with insights into their daily struggles and successes. The second focus group consisted of two educational advisors and one lean coach who coached several nursing teams during the introduction of differentiated nursing practices. They were invited to share their roles' challenging and positive aspects, which we then explored further. This helped us to better understand how they gave meaning to differentiated nursing practices in their daily work.

#### 4.4.5 Podcast

A podcast series of seven 50‐min episodes was recorded after a first analysis of the data. In each episode, a member of the inside research team and two ‘guests’ from inside the hospital were invited to reflect on different themes deemed important for advancing differentiated nursing practices. Each episode was hosted by a member of the outside research team [DM, HS and PL]. By placing participants with different backgrounds together at the podcast table, we stimulated rich conversations about the future of the nursing profession and differentiated nursing practices.

### 4.5 Data analysis

Data analysis took place in two stages. First, in October 2020, a selection of experts from inside and outside the hospital were invited to participate in a full‐day data analysis session. The outside experts were a professor (emeritus) of management, a professor of nursing science and a lecturer of ethics in care. They were experts on organizational change processes and/or developments within the nursing profession. The inside experts were a nurse scientist and the project lead responsible for implementing differentiated nursing practices. They were experts on the implementation process of differentiated nursing practices in the hospital and/or in the field of qualitative research. Anonymized transcripts of the interviews were divided among half the participants of the session beforehand. These participants shared their reflections and associations with the transcripts during the data‐analysis session. The other participants were invited to react to these reflections and were asked to relate them to their own knowledge and expertise. All members of the analysis session discussed the findings and identified themes together. The selected themes were all aimed towards answering the research question that had been formulated and aimed at organizational learning and change. As such, a shared story could be created to which all organizational members could relate (Roth & Kleiner, 1995). This process led to a written learning history (Martini et al., 2021b) and a thematic podcast series (Martini et al., 2021a of seven episodes in which differentiated nursing practices and the future of the nursing profession of the hospital were addressed. The written learning history and podcast were shared both in and outside hospital boundaries, as they are most effective when broadly shared and discussed (Bradbury et al., 2015, p. 23).

In the second stage (May, June and July 2023), we [DM, PL] revisited the data to further sharpen the initial thematic ordering. In this stage, we included the podcast recordings. We uploaded the recordings of the podcast episodes in Atlas.ti, which incorporates the ability to listen and code audio data. Data were gathered and analysed concurrently, with analysis unfolding gradually across the various research phases. Use of different data collection methods helped us gain a richer insight into the practices concerning enacting new nursing roles (Coté‐Arsenault, 2013; Lambert & Loiselle, 2008). For example, in the interviews and focus groups, we could dive deeper into the reasons why individuals embraced or resisted the role of nurse coordinator, while, during shadowing, we could observe group dynamics and ‘raw’ emotions concerning the new role. The data were gathered and analysed iteratively and moved between data from interviews, focus groups, documents, shadowing and podcasts. The triangulation of the different data enhanced our understanding of enacting new nursing roles (Lambert & Loiselle, 2008). We [DM, HS, PL, LS and MN] continuously discussed and compared our findings in relation to the literature. Differences in opinion were used to discuss how the ideas generated by the research might impact nurses' daily practices and vice versa. This is in line with *interpretive description* in which the purpose is not to codify ‘universal truths’ but rather to know how to apply them in different unique cases (Thorne, 2016).

### 4.6 Ethical considerations

This study obtained ethical approval (CMO 2019‐5992). All participants were guaranteed confidentiality and written approval was obtained. Participation was voluntary, and participants were fully informed of the study before giving their consent. They had the right to withdraw at any time. Participants featured in the podcast were allowed to review and, if desired, make edits or request the removal of their contributions before the podcast's launch. All data were stored under identification numbers according to scientific rules and legislation.

## 5 RESULTS

Our findings reveal how roles of nurse coordinators were enacted and negotiated within nursing teams and in the hospital. Previous attempts to differentiate were taken into account, and proponents of differentiated nursing practices worked to change nurses' narratives as all equal and positioned at the bedside by discursively expanding the meaning of nursing work. Three themes emerged that tell the story of the process of reconfiguring nursing practices: (1) stretching the nature of ‘nursing work’, (2) using earlier experiences and (3) collectively tackling taboos.

### 5.1 Stretching the nature of nursing work

This theme reveals how proponents of differentiated nursing practices aimed to create spaces to negotiate and enact new nursing roles. They did this by stretching the perceived nature of nursing work.

Nursing work involved delivering high‐quality patient care and representing the patients' interests within a multidisciplinary environment. The main sentiment in nursing teams was that the work centred around a patient's bedside and focused on practical and technical skills. Other tasks were, for the most part, not defined as work. This question to an aspiring nurse coordinator illustrates, *Are you working today, or will you be in your office?* However, aspiring nurse coordinators saw nursing work as more than direct patient care. They emphasized during interviews that quality improvement, coaching or research should be seen as nursing work too as it was needed to improve the quality of patient care and the position of nurses. They hoped that their teams could achieve a situation in which they *could be proud to call ourselves a nurse even though we are not at a patient*'*s bedside every day*.

Stretching the meaning of nursing work was perceived as difficult: *I still have to fight for time away from the bedside. If it's quiet, and I withdraw from direct care, I will have colleagues scold me for not taking care of my patients*. *You are on the list, so you have to work* (*aspiring nurse coordinator*). Being assigned patients meant you remained close to them. Even when it was quiet, a patient might need you. Because of this strong focus on direct patient care as the core business of nursing work, some nurse coordinators encountered resistance; *Your patients need bathing, and that wound needs to be taken care of. So stop harassing us with your research* (*An aspiring nurse coordinator shared how her colleagues feel about her new role*).

Although participants were explicit in their interviews about stretching nursing work to legitimize their new role, they were worried: *I fear that my new role will limit itself to supervising daily practices which, for me, is not enough* (*Aspiring nurse coordinator*). Being appointed as nurse coordinators would act as legitimization to shape their new roles. *Once I am officially a nurse coordinator, […] we can start to talk about how to further shape our role* (*aspiring nurse coordinator*). However, the hospital had ample experience with differentiated nursing practices. Different forms (task differentiation, the introduction of nursing aids, senior roles, etc.) have been tried, introduced or cast aside for decades. These earlier attempts to differentiate showed that being appointed in a new role did not mean that others would change their views on nursing work. For example, when the first bachelor‐trained nurses entered the hospital in the 1980s (introduced as future leaders who could transform nursing), they were badly received by their nursing colleagues: *We were loathed by other nurses. We couldn't do anything, and we didn't know anything*. (*bachelor‐trained nurse's reflection on the start of her career as one of the first graduates of the bachelor training*). Strong sentiments against differentiated nursing practices and change were expressed in nursing wards and the hospital organization. ‘*They* (*the hospital*) *decide this for us. They try something new every five years*’ (*nurse*). Resistance was fuelled by earlier attempts to differentiate. This complicated stretching nursing work and enacting new roles. As such, earlier experiences indicated that relying on an official new job title would not help nurses to further shape their new roles.

### 5.2 Using earlier experiences

This theme reveals that earlier experiences with implementing differentiated nursing practices and change influenced the current attempt to differentiate. Also, it shows the importance of using these experiences.

*Looking at where we started a few years ago and where we are today, I think we really tried to involve everyone in the process. To see differentiated nursing practices as an effort towards team development instead of it being the goal in itself* (*manager*).

This quote shows that the hospital had learned from earlier attempts to introduce differentiated nursing practices. In 2016, for example, the hospital initiated an attempt to differentiate. It aimed to develop and improve patient care continuously from a nursing perspective. Its focus was *to* ‘*utilize the qualities of all nursing professionals, regardless of* (*initial*) *training or specialization*’ (*programme manager*). The new role of nurse coordinator would be added to nursing teams. A top‐down training programme supported nurses in their development. Generic training on nursing leadership, evidence‐based practices, clinical reasoning and patient‐centred care was offered to all nurses. The training was separated into sessions for bachelor‐trained nurses and vocationally trained nurses. After this training, the bachelor‐trained nurses were expected to shape their new roles as nurse coordinators in practice within their nursing teams. The training programme was badly received: ‘*Nurses that do not qualify as nurse coordinators feel devaluated*’ (*educational advisor*). Furthermore, bachelor‐trained nurses felt they were thrown into the deep end when shaping their new roles in daily practices. Implementation of the new role was complicated. As such, the hospitals' attempt to incorporate the qualities of all nursing professionals, regardless of (initial) training, had split nursing teams into two groups and created stress among nurses.

Based on the critique, the original programme was adapted in 2019 to meet team needs with a special focus on team goals. Nursing teams were allocated time to practice new roles, coached by an educational advisor. The hospital used earlier experiences to alter its programme. From top‐down to bottom‐up, and from individually driven to team driven. However, resistance remained, and nurses still felt forced to cooperate in the programme: *The hospital board and the nursing council are very positive about the new amendment, so we do not really have a choice but to play along*’\ (*nurse*). Still, once nurses became directly involved in the new programme, they had some positive experiences. *As it is presented today, the program is much better than it was. Because there is a focus on team coaching, for example. I see why it can be valuable for our team* (*nurse*). We saw how the new programme got the benefit of the doubt. Slowly, teams started to feel more comfortable about the introduction of new roles.

However, earlier experiences also influenced how nurses acted in the new programme as one educational advisor acknowledged: *When I start with a team, I try to get them to focus on the future. To encourage a team's shared ambitions to improve patient care. But people often find this difficult. They feel that their ambitions might fall outside their scope of influence. They will say* ‘*We have tried this before and it did not work*’. *So, to get nurses to believe that they can actually influence their own work in this new program is a challenge* (*educational advisor*).

As such, the educational advisors reflected on such experiences with nurses, and together, they aimed to find new ways to become more influential.

### 5.3 Collectively tackling taboos

*If we have to start with this program again, so be it. But I do not want to hear the term ‘differentiation’ mentioned in my team. We are under enough pressure as it is* (*middle manager of a nursing ward*). ‘Differentiation’ was a loaded term in the hospital for many nurses and managers. For this middle manager, mentioning it was taboo. She worried it would negatively affect her team. Her worries were based on what she experienced in 2016 during the former attempt to differentiate. Differentiating between nurses based on their initial training level was a sensitive topic throughout the hospital. All the questions I get asked about the new program come down to how to avoid offending nurses in our hospital (manager). This was at odds with other specialized roles for nurses which they trained for *after* their initial training programme. *Everyone agrees that a nurse without special training cannot work in the Intensive Care Unit. But if you have completed a bachelor's in nursing, and you are adept at and willing to perform the tasks of the nurse coordinator, it suddenly becomes a big problem because everyone has to be equal* (*board member*). In this case, however, the role of *the nurse coordinator was directly linked to the question of whether* nurses were allowed to be different based on their initial training level. This appeared difficult not only for nurses to talk about but also for other participants. The participants who spoke about new nursing roles added disclaimers such as *But I would not want anyone to think that I do not respect or appreciate the hard work that all nurses do*’ (*physician*) or ‘*But of course, we all want to remain equal in our team* (*aspiring nurse coordinator*).

The new approach that started in 2019, after the amendment to differentiate was abolished, affected the taboo that rested on differentiated nursing practices and the role of the *nurse coordinator*. Instead of focusing on individual nurses who were supposed to fulfil the role of nurse coordinator, it shifted to a team‐oriented approach in which differentiation was linked to team goals instead of new roles. This meant that team members had to work together to achieve these goals instead of being separated into two groups. As such, spaces appeared where it became possible to discuss new nursing roles. *When we started in 2016, the programme played a central role in the division of bachelor‐ and vocationally‐trained nurses. And that has changed into more of a team development trajectory. The entire team is stimulated, and that is what really resonates with the teams right now* (*aspiring nurse coordinator*).

In the new programme, teams formulated team goals to improve patient care and different nursing roles (nurse, nurse coordinator, specialized nurse, etc.) were matched to achieve these goals. Teams were not required to include the nurse coordinator role in their improvement plans. However, most teams opted for the inclusion of nurse coordinators. This affected how differentiated nursing practices were perceived. *When it is no longer about whether you can or cannot be a nurse coordinator and more about who we need to achieve this goal, it takes the heat out of the debate* (*educational advisor*). Instead of emphasizing the qualities of the nurse coordinators, both the technical and relational skills of all nurses in the team became point of focus.

*All team members have their own characters and their own qualities. And that is beneficial to our team. […] I believe that when people get the chance to do what they like, and what they excel in, this translates to good quality healthcare* (*aspiring nurse coordinator*). This new collective responsibility led to more openness towards differentiated nursing practices. *My wish for our team is that nurses can specialize in, for example, research or coaching and combine that with direct patient care in our nursing ward. We remain one team, and we are all of equal importance, but we can make use of each other's qualities* (*aspiring nurse coordinator*). The hospital had learned from previous attempts to differentiate. Making nurse coordinators a means to achieve team goals, instead of the main goal, slowly provided nurses with comfortable spaces to speak about the role of nurse coordinator, and through that, slowly tackled the long‐lasting taboo.

## 6 DISCUSSION

This study aimed to better understand the enactment of new future‐oriented nursing roles in a general hospital by acknowledging its complex socio‐historical context. Our analysis reveals that enacting the role of nurse coordinator was closely linked to and influenced by the specific local and historical context into which the role was introduced. This became apparent in three themes that describe how spaces were created into which the new role was enacted. First, by stretching the nature of nursing work; second, by using earlier experiences; and third, by collectively tackling taboos.

Our first key finding shows how advocates of the nurse coordinator role aimed to create spaces for this role by discursively stretching the meaning of nursing work to include other work than direct patient care. These findings relate to the literature regarding efforts of, for example, other healthcare professionals challenging stereotypical images by denouncing traditional professional values and discursively reframing themselves as team players who work across disciplinary and organizational boundaries (Berghout et al., 2018, p. 73). However, stretching the nature of nursing work was complicated because of persistent historically formed images of nursing as ‘bedside work’. In line with earlier research findings, we saw how these images hampered the positioning of new nursing roles in healthcare organizations (Matthias, 2015; Rafferty, 2018). As such, we emphasize that these images are not just ‘stories’ but shape nursing identities (Oldenhof et al., 2016, p. 52; Verhoeven, Van de Loo, et al., 2023). They deny the value of the unique qualities of individual nurses (Van Der Cingel & Brouwer, 2021).

Using earlier experiences was a second main finding in the study. We showed that earlier top‐down attempts to introduce the nurse coordinator role in nursing teams in the hospital had split nursing teams into two groups and created stress among nurses. In addition, nurses found it difficult to initiate changes based on earlier experiences in which their attempts to exert influence had failed. A new bottom‐up approach that focused on the needs and wishes of nursing teams instead of the introduction of the role of nurse coordinator appeared to be better received. This is in line with earlier research that argues that the introduction of new nursing roles is a collective effort (Martini et al., 2023; Torrens et al., 2020) that requires a bottom‐up approach in which nurses are allowed to experiment based on action and appraisal (Van Schothorst–van Roekel et al., 2021, p. 8). In addition, it shows the importance of acknowledging and addressing controversial histories by reflecting on and learning from them (Smith, 2020).

Thirdly, we found that it was important to tackle taboos collectively. Shifting attention from individual nursing roles to relations and goals in nursing teams created spaces where new nursing roles became negotiable. We show – in line with Torrens et al. (2020) – that when new roles are introduced, negotiation of these new roles between team members is key to a successful implementation. These negotiations are ‘*an opportunity to reflect on current practices and models of care as to establish a shared vision for the team*’ Torrens et al. (2020). In such instances, nurses engage with others to create new meanings or directions in their work. The literature describes them as ‘leadership moments’, that is, collective acts of empowerment (Ladkin, 2020). This study underlines the importance of recognizing that acts of empowerment are relational processes that must be fostered collectively (Ladkin, 2020; Martini et al., 2023; Verhoeven, Marres, et al., 2023; Verhoeven, Van de Loo, et al., 2023).

The three interconnected themes show the importance of addressing particular sociohistorical contexts when introducing new nursing roles. We show that enacting a nurse coordinator role was influenced by contextual factors that varied from ward to ward and from nursing team to nursing team. Furthermore, (historically shaped) assumptions about the nature of nursing work, for example, or specific experiences with earlier attempts to differentiate, affected how the new role was enacted. The negative effects of a Dutch legislative proposal to define differentiation in nurse staffing by law in 2019 (see Felder et al., 2022; Schalkwijk et al., 2024) made clear that nurses need to be involved in decision‐making processes concerning their profession (Van Kraaij et al., 2021). However, the legislation and the complex history of differentiated nursing practices (Matthias, 2015), linked to a strong narrative of nursing as ‘bedside work’ (Felder et al., 2022; Rafferty, 2018), led to a taboo on mentioning differentiated nursing practices in nursing teams. We found that reflecting upon earlier experiences and using them to improve current efforts to introduce new nursing roles helped to soften strong (historically) grown emotions (Smith, 2020).

This resonates with research underscoring that it is vital to better understand and consider contexts in light of changes in nurse staffing and differentiated nursing practices (Griffiths, 2014; Van Schothorst–van Roekel et al., 2021). More research is conducted on contextual factors that help or hinder change (e.g. see Cammer et al., 2013; Squires et al., 2019). However, as contexts are unstable, unfolding processes (May et al., 2016), capturing them in general ‘factors’ might not be sufficient. Zooming in on the daily practices in which new nursing roles are introduced can help us better understand how change might be accomplished. Moreover, our findings indicate that using these daily practices *as starting points* for change can be beneficial when introducing new nursing roles.

## 7 STRENGTHS AND LIMITATIONS

The multiple data‐gathering methods gave us a unique understanding of differentiated nursing practices from which we derived a broader relevance (Coté‐Arsenault, 2013; Lambert & Loiselle, 2008). However, this study has some spatial and temporal limitations to consider. First, although nurse staffing and differentiated nursing practices are international topics of interest (Boston‐Fleischhauer, 2019; Carryer, 2019; Matthias, 2015), the findings of this study are to be understood within the specific Dutch context in which the study took place. This potentially limits the ability to generalize our findings to other contexts. Second, this study represents the findings collected at a specific moment while introducing differentiated nursing practices is a dynamic process that continues to develop over time.

## 8 RECOMMENDATIONS FOR FURTHER RESEARCH

Further research, both longitudinal and international, on how the introduction of new nursing roles plays out in different contexts over time is recommended.

## 9 IMPLICATIONS FOR POLICY AND PRACTICE

Our findings indicate that when introducing differentiated nursing practices, hospitals should use local, situated conditions as starting points for introducing new nursing roles. Furthermore, shifting focus from individual nursing roles to nursing team development softens strong (historically) grown emotions and creates comfortable spaces in which new roles become negotiable. From a more practical point of view, helping an organization reflect on its history, for example, with the learning history method, helps to create spaces for new nursing roles and to stretch the nature of nursing work. Furthermore, this study shows that investing in a programme that supports nurses in enacting new roles and reshaping their daily practices is important. This is a collective responsibility of both nurses and the hospital organization (Martini et al., 2023; Verhoeven, Marres, et al., 2023).

## 10 CONCLUSION

Nursing shortages call for healthcare organizations where nurses can fulfil roles attuned to their educational backgrounds and experiences. This includes roles explicitly designed for bachelor‐trained nurses. However, differentiated nursing practices, nurse staffing and skill mixes have a long and complex history that triggers emotions. This study shows how proponents negotiated the new nurse coordinator role in nursing teams. With a bottom‐up approach, focused on collective responsibilities, and by acknowledging and reflecting on the past, spaces were enacted in which the role of nurse coordinator became one role, among others, in the delivery of patient care. When changes in the nursing profession evoke resistance or emotion, this study shows that it is important to get close to daily practices, open up (earlier) collective experiences, acknowledge taboos and search for alternatives.

## AUTHOR CONTRIBUTIONS

All authors have agreed on the final version and meet at least one of the following criteria (recommended by the ICMJE*): (1) substantial contributions to conception and design, acquisition of data or analysis and interpretation of data; (2) drafting the article or revising it critically for important intellectual content.

## FUNDING INFORMATION

This study is part of the Dutch National Research Consortium RN2Blend and is funded by the Dutch Ministry of Health, Education and Sports.

## CONFLICT OF INTEREST STATEMENT

The authors declare no conflict of interest.

### PEER REVIEW

The peer review history for this article is available at https://www.webofscience.com/api/gateway/wos/peer‐review/10.1111/jan.16240.

## Data Availability

The data that support the findings of this study are available on request from the corresponding author. The data are not publicly available due to privacy or ethical restrictions.
